# Validation of the Mycelium Transfer Method Coupled with MALDI-TOF MS for Rapid Identification of Filamentous Fungi

**DOI:** 10.3390/pathogens15090992

**Published:** 2026-09-19

**Authors:** Ruchika Bagga, Johan Delport

**Affiliations:** 1 Department of Pathology and Laboratory Medicine, London Health Sciences Centre, London, ON N6A 5W9, Canada; 2 Schulich School of Medicine & Dentistry, Western University, London, ON N6A 3K7, Canada; 3 Division of Microbiology, London Health Sciences Centre, London, ON N6A 5W9, Canada

**Keywords:** MALDI-TOF MS, filamentous fungi, mycelium transfer, triplicate spotting, *Aspergillus*, Mucorales, dermatophytes, clinical mycology, diagnostic mycology, fungal proteomics, CLSI M52

## Abstract

**Background:** Invasive filamentous fungal infections carry substantial mortality among immunocompromised hosts, and diagnostic delay associated with morphology-based mould identification is a recognised contributor to poor outcomes. The Mycelium Transfer (MyT) method, implemented on the Bruker MALDI Biotyper^®^ platform, allows direct mass spectrometric profiling of leading-edge mycelium sampled from routine solid agar without solvent extraction. We conducted a single-centre analytical validation of an optimised, triplicate-spot MyT workflow as a standalone identification platform at a Canadian tertiary care academic centre. **Methods:** Fifty-six archived, pre-characterised clinical isolates spanning five fungal categories—*Aspergillus* species (n = 21), other hyaline non-*Aspergillus* moulds (n = 14), Mucorales (n = 8), dermatophytes (n = 8), and dematiaceous fungi (n = 5). These isolates were selected to represent our local isolate population and tested by MyT on a Bruker Microflex LT/SH system, with acquisition and scoring performed prospectively by technologists blinded to the archived reference identification. Spectra were interrogated against the MBT Myco 4.1 Filamentous Fungi Library. Each isolate was spotted in triplicate; a final identification required ≥2/3 concordant spots at a score ≥ 1.8, consistent with CLSI M58 guidance. A parallel formic acid–ethanol extraction was performed for a subset of 11 isolates as an exploratory, within-study comparator. Primary culture media were Brain Heart Infusion (BHI) agar and Sabouraud Dextrose Agar with chloramphenicol and gentamicin (SAB-CG). Agreement was assessed against CLSI M52 criteria, with an a priori target of ≥90% overall concordance. **Results:** Applying the triplicate-spot consensus rule at the ≥1.8 threshold, species- or complex-level identification was achieved for 43/56 isolates (76.8%; 95% CI 64.2–85.9%), and overall acceptable identification (species/complex plus acceptable genus-level calls) for 50/56 isolates (89.3%; 95% CI 78.5–95.0%). No misidentifications were observed. *Aspergillus* spp. and dermatophytes each achieved 100% overall identification; performance was lowest for dematiaceous fungi (2/5, 40.0%). In a post hoc analysis restricted to the categories in which discordant single-spot results occurred (other hyaline non-*Aspergillus* moulds and Mucorales, combined n = 22), triplicate consensus corrected three isolates in which a single high-scoring spot would have produced a false identification, and enabled identification of four further isolates that a single spot would have missed, corresponding to an estimated single-spot equivalent identification rate of 12/22 (54.5%) versus 19/22 (86.4%) with triplicate consensus, which is an approximately 32 percentage point absolute difference restricted to this 22-isolate subgroup and not an effect demonstrated across the complete 56-isolate panel. Growth sufficient for MyT sampling at 72 h was numerically higher on BHI agar than SAB-CG for hyaline moulds at 72 h (88.6% vs. 77.1%), although this difference was not statistically significant (McNemar’s test, *p* = 0.29). In a smaller exploratory subset of 11 isolates tested in parallel by both methods, MyT showed similar performance to formic acid–ethanol extraction (equivalent or higher scores in 8/11 isolates tested in parallel); this was not a formally powered non-inferiority comparison. **Conclusions:** A triplicate-spot MyT workflow at a ≥1.8 score threshold achieved 89.3% overall concordance, with reference identification and zero misidentifications across a 56-isolate panel, approaching the CLSI M52 ≥ 90% benchmark overall and meeting it for *Aspergillus* species and dermatophytes specifically. To our knowledge, this is among the first Canadian evaluations of the MyT method for routine clinical mould identification. Dematiaceous fungi and several individual species within other categories remained underpowered and of limited performance, reflecting both small subgroup sizes and known gaps in current spectral library coverage. These findings support MyT as a practical frontline identification method for *Aspergillus*, Mucorales, and dermatophytes within existing laboratory infrastructure, with chemical extraction or molecular methods retained as adjuncts for isolates that do not achieve threshold identification; broader implementation and clinical outcome benefits remain to be evaluated prospectively.

## 1. Introduction

Invasive filamentous fungal infections are a major cause of morbidity and mortality among immunocompromised patients, including recipients of haematopoietic stem cell and solid organ transplants, individuals receiving prolonged high-dose corticosteroid or immunosuppressive therapy, and patients with haematologic malignancies undergoing induction chemotherapy. Case fatality rates for invasive aspergillosis range from 30 to 70%, despite guideline-concordant antifungal therapy, while mucormycosis and fusariosis are associated with mortality exceeding 50% in the most vulnerable hosts [1,2]. The rising incidence of invasive fungal disease, emerging antifungal resistance, and resource constraints in diagnostic mycology services have been identified as priorities for global public health action [3].

A modifiable contributor to poor outcomes is diagnostic delay. Conventional identification relies on macroscopic colony morphology, microscopic examination of conidial and hyphal structures, and dimorphism studies where applicable. This approach is time consuming and can be difficult to apply consistently: closely related genera frequently share overlapping macroscopic and microscopic features, isolates may be atypical or incompletely sporulated, and reliable interpretation depends on technologist expertise that requires years of dedicated mycology training to develop. As a result, morphology-based mould identification often requires days to weeks and is subject to inter-observer variability [4]. Cohort studies have shown that time-to-species-directed antifungal therapy is independently associated with survival in invasive mould infections, such that each additional day of diagnostic delay may narrow the window during which therapy can alter the outcome [5]. These limitations underscore the critical need to modernise fungal diagnostics in routine clinical laboratories by developing rapid, accurate, and scalable identification approaches that reduce diagnostic delay, improve species-level resolution, and support earlier evidence-based antifungal therapy.

Matrix-assisted laser desorption/ionisation time-of-flight mass spectrometry (MALDI-TOF MS) has transformed bacterial and yeast identification since its introduction into clinical laboratories in the mid-2000s, offering species-level accuracy within minutes at low per test cost, and remains a mainstay of routine clinical microbiology diagnostics [6,7]. Extension of this technology to filamentous fungi has been considerably more difficult, for three interrelated reasons. First, the rigid, chitinous cell walls of mould hyphae resist the disruption protocols that are adequate for bacteria and yeasts, impeding protein extraction and spectral signal generation; conventional formic acid–ethanol extraction protocols were developed to overcome this barrier but add substantial hands-on time and technical complexity to routine workflows [8]. Second, the proteomic profile of a mould colony varies across developmental stages, such that conidia, leading-edge hyphae, aerial mycelium, and sporulated surface material can yield qualitatively different mass spectra, introducing intra-isolate variability that can undermine library matching. Third, commercial spectral library coverage for filamentous fungi has historically been more limited than for bacteria and yeasts, particularly for less common and melanised species [9]. Contemporary multi-platform and multi-library comparisons continue to report substantial variability in identification yield across institutions and pre-analytical protocols, underscoring that sample preparation strategy and library currency, rather than instrumentation alone, remain key determinants of diagnostic performance for filamentous fungi [5,10].

The Bruker MBT HT Filamentous Fungi Module, incorporating the Mycelium Transfer (MyT) workflow, addresses the first two of these challenges directly and, when combined with an optimised acquisition strategy, can partly mitigate the third challenge, as well. Mould mass spectra can show spot-to-spot variability attributable to micro-heterogeneity in the harvested mycelial material, such that a single acquisition may not be a reliable basis for a definitive identification. Triplicate spotting with a ≥2/3 concordance rule at the approved ≥1.8 threshold has been proposed as a means of mitigating this variability, although the magnitude of this benefit specifically within the MyT workflow has not been well characterised.

This paper describes a single-centre analytical validation of the MyT method, used as a standalone platform, on the Bruker Microflex LT/SH system, using an archived isolate panel purposively assembled to represent the taxonomic categories relevant to LHSC’s patient population. The primary objective was to determine whether an optimised triplicate-spot MyT workflow achieves ≥90% overall concordance with reference identification per CLSI M52. Secondary objectives were to characterise performance by fungal category, culture medium, and incubation duration, to quantify the contribution of triplicate spotting over single-spot acquisition, and to compare MyT with formic acid–ethanol extraction in a subset of isolates.

## 2. Materials and Methods

### 2.1. Study Setting and Ethics

This study was conducted in the Microbiology Department at London Health Sciences Centre (LHSC), London, Ontario, Canada. All procedures were conducted in accordance with institutional laboratory protocols. Formal Research Ethics Board review was not required under the Canadian Tri Council Policy Statement (TCPS 2) criteria for laboratory validation studies using non-identifiable residual clinical material; further detail is provided in the Institutional Review Board Statement.

### 2.2. Isolate Panel and Reference Identification

This was a single-centre analytical (technical) validation, not a prospective clinical validation study. The isolates evaluated were archived rather than consecutively collected fresh clinical specimens, while MyT testing, spot scoring, and analysis were performed prospectively, in real time, by technologists blinded to the archived reference identification during acquisition. Isolate selection was purposive rather than consecutive, and isolates were drawn from the −80 °C frozen archive and the IQMH proficiency testing collection specifically to ensure representation of all five taxonomic categories relevant to our patient population, with deliberate over representation of *Aspergillus* species given their clinical prevalence in the local oncology and transplant patient population.

The validation panel comprised isolates from two sources: (i) clinical isolates for which the original reference identification was established by a reference laboratory using a combination of macroscopic/microscopic morphology with molecular sequencing (ITS, β-tubulin, or calmodulin gene targets, as applicable) performed selectively for isolates that were morphologically ambiguous, and (ii) previously characterised proficiency testing isolates from the Institute for Quality Management in Healthcare (IQMH) external quality assessment programme, for which the target identification is established through the programme’s inter-laboratory consensus and/or molecular reference testing. Molecular confirmation was not performed uniformly for every isolate in the panel, which we acknowledge as a limitation in Section 5.

The panel comprised: (1) *Aspergillus* species (*A. fumigatus*, *A. flavus*, *A. niger*, *A. nidulans*, *A. terreus*, *A. versicolor* complex, *A. ustus* complex; n = 21); (2) other hyaline non-*Aspergillus* moulds (*Fusarium solani* complex, *Purpureocillium lilacinum*, *Scedosporium apiospermum*, *Lomentospora prolificans*, *Byssochlamys spectabilis*, *Beauveria bassiana*, *Rasamsonia argillacea*, *Sporothrix schenckii*, *Scopulariopsis* spp.; n = 14); (3) Mucorales (*Mucor circinelloides*, *Rhizopus arrhizus*, *Rhizopus microsporus*, *Lichtheimia corymbifera*, *Cunninghamella bertholletiae*; n = 8); (4) dermatophytes (*Trichophyton mentagrophytes* group, *T. rubrum* group, *T. tonsurans*, *Nannizzia gypsea*, *Microsporum canis*, *Epidermophyton floccosum*; n = 8); (5) dematiaceous fungi (*Exophiala jeanselmei*, *Alternaria chartarum*, *Bipolaris* spp., *Cladosporium* spp.; n = 5). Quality control strains (*Aspergillus brasiliensis* ATCC 16404 and *Trichophyton interdigitale* ATCC 9533) were run at the commencement of each testing day.

We note that, with the exception of *A. fumigatus* and *A. niger*, most individual species and species complexes within this panel are represented by fewer than 3 isolates, and several by a single isolate. Category-level concordance estimates should therefore be interpreted as the primary performance measure of this validation, with species-level findings regarded as preliminary and hypothesis generating (Section 5).

### 2.3. Culture and Subculture Conditions

Archived isolates were recovered from −80 °C Microbank^®^ storage and subcultured in parallel onto two routine solid media: Brain Heart Infusion agar with 5% sheep blood, chloramphenicol, and gentamicin (Thermofisher Scientific, Waltham, MA, USA) and Sabouraud Dextrose Agar with chloramphenicol and gentamicin (SAB-CG; Thermofisher Scientific, Waltham, MA, USA). All culture setup and MALDI-TOF MS sample preparation was performed within a Class II biological safety cabinet. Primary evaluation was performed at 72 h post subculture (Day 3); dermatophytes were additionally evaluated at 96 h (Day 4) because of their slower growth. For the *Aspergillus* and other hyaline non-*Aspergillus* categories, a secondary harvest and MyT acquisition was additionally performed at 168 h (Day 7) on the subset of isolates from these categories with sufficient remaining growth for re-sampling in order to assess the effect of advancing aerial conidiation on spectral performance; this Day 3 versus Day 7 comparison (Section 3.8) was a paired, descriptive, exploratory comparison and was not a pre-registered hypothesis test. Mucorales were tested only at Day 3 because of the risk of colony overgrowth beyond this timepoint.

MyT testing throughout the study was performed by 2 trained technologists; a formal inter-operator reproducibility assessment was not performed and is discussed as a limitation in Section 5.

### 2.4. MyT Sample Preparation and MALDI-TOF MS Acquisition

**Figure 1:**
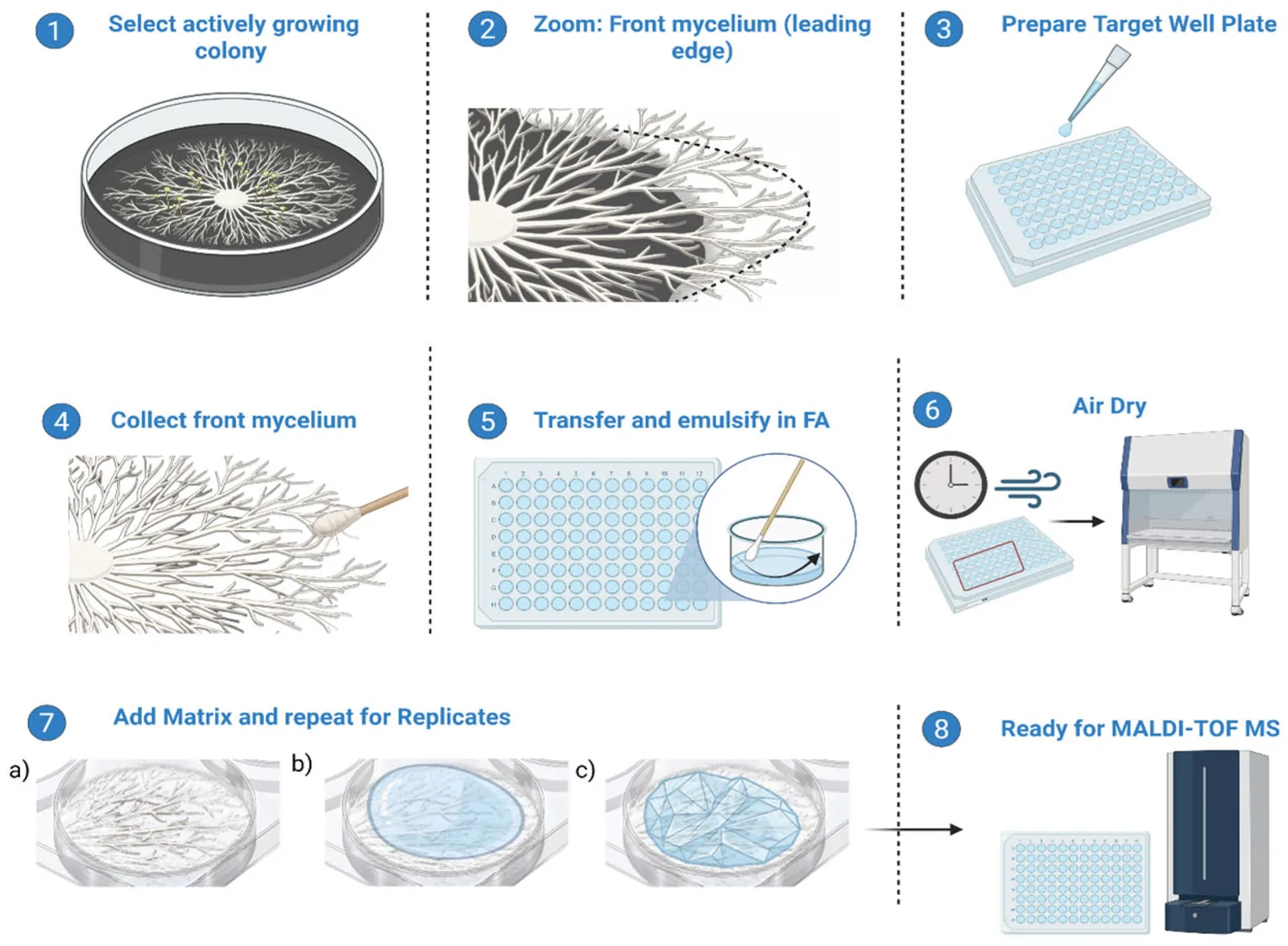
Schematic of the Mycelium Transfer (MyT) sampling method, illustrating harvest of leading-edge mycelium from the colony periphery.

The MyT procedure was performed as described by the manufacturer [11] and standardised for routine use at LHSC. For each isolate, 1 μL of 70% formic acid (Bruker Daltonics) was dispensed onto three separate wells of an MBT Biotarget 96 plate. A disposable sterile wooden inoculation stick was lightly moistened by contact with the formic acid droplet, then drawn across the actively growing, non-sporulated leading edge of the colony using minimal pressure to harvest undifferentiated hyphal material without penetrating the agar (Figure 1). The harvested material was transferred to the target well and emulsified by circular motion within the formic acid droplet; this was repeated with a fresh stick for each of the three replicate wells. After air drying within the biosafety cabinet (approximately 2–3 min), 1 μL of HCCA matrix solution was overlaid and allowed to co-crystallise. The target plate was analysed on the Bruker Microflex LT/SH system using MBT Compass HT software version 2023 in Filamentous Fungi mode.

Spectra were acquired over the mass range 2000–20,000 Da and interrogated against the Bruker MBT Myco v4.1 Filamentous Fungi Library. A final identification was accepted when ≥2 of 3 replicate spots achieved a score ≥ 1.8 with a concordant organism-level designation. Identifications were recorded at the species level where possible; complex-level designations were used where the library could not resolve individual cryptic species within a complex.

For a subset of 11 isolates, spanning the *Aspergillus*, Mucorales, dermatophyte, dematiaceous, and other hyaline categories, a parallel formic acid–ethanol extraction procedure was performed as an exploratory, within-study comparator to give an initial indication of how MyT performance compares with a conventional extraction-based sample preparation method; this was not a formally powered non-inferiority comparison (Section 3.10). Briefly, a 1 cm^3^ block of mycelium and agar was excised from the leading colony edge, suspended in 300 μL absolute ethanol, vortexed, and centrifuged (13,000× *g*, 2 min); the supernatant was discarded and the pellet air dried before addition of 50 μL 70% formic acid and 50 μL acetonitrile, with a further centrifugation step (13,000× *g*, 2 min); 1 μL of the resulting supernatant was spotted in triplicate onto the target plate and overlaid with HCCA matrix. Extracted spots were interrogated against the same MBT Myco 4.1 library using identical threshold and concordance criteria as the MyT spots.

### 2.5. Identification Criteria and Score Threshold

The score threshold for mould identification was set at ≥1.8 across all categories, consistent with the Bruker MBT HT Filamentous Fungi Module specification and CLSI M58 guidance for mould identification by MALDI-TOF MS [12,13]. Scores below 1.8 were classified as no identification, regardless of proximity to the threshold. Identification outcomes were classified as: Correct Identification (CI)—match to the reference identification at species or species-complex level; Acceptable Identification (AI)—genus-level match where species-level resolution was not achieved; Misidentification (MI)—an incorrect genus assigned; Unidentified (UI)—no replicate spot achieved a score ≥ 1.8.

### 2.6. Statistical Analysis

Identification rates were summarised descriptively by fungal category, culture medium, and incubation duration, with 95% confidence intervals for proportions calculated using the Wilson score method. Score distributions across triplicate spots are reported as mean ± standard deviation. Paired identification outcomes on BHI versus SAB-CG at Day 3 were compared using McNemar’s test for paired binary data; a two-sided *p*-value < 0.05 was considered statistically significant. The triplicate- versus single-spot comparison (Section 3.9) and the Day 3 versus Day 7 comparison (Section 3.8) were post hoc, exploratory analyses defined after data collection and are reported as such, without formal correction for multiple comparisons. Given the modest sample size within individual fungal categories, all subgroup estimates should be interpreted as descriptive rather than definitive, as reflected in the width of the corresponding confidence intervals. Analyses were performed in Microsoft Excel (Microsoft Corporation, Redmond, WA, USA), with confidence intervals calculated separately in Python 3 (SciPy).

## 3. Results

### 3.1. Panel Composition and Overall Performance

Fifty-six pre-characterised isolates were evaluated across five fungal categories (Table 1). No misidentifications were recorded in any category. Using the triplicate-spot consensus rule at the ≥1.8 threshold, correct identification at the species or species complex level was achieved for 43/56 isolates (76.8%; 95% CI 64.2–85.9%). Including acceptable genus-level identifications, overall concordance was 89.3% (50/56; 95% CI 78.5–95.0%), approaching but not formally meeting the CLSI M52 ≥ 90% passing threshold for the panel as a whole; the threshold was met individually by the *Aspergillus* and dermatophyte categories. Six isolates (10.7%) yielded no identification across all three replicate spots; five of these were dematiaceous fungi or rare moulds underrepresented in the reference library, suggesting that library composition, rather than the MyT sampling method itself, was the primary limiting factor for this subset.

### 3.2. Aspergillus Species (n = 21)

*Aspergillus* species showed the strongest performance of any category evaluated. All 21 isolates (100%; 95% CI 84.5–100%) achieved overall identification at ≥1.8 (correct genus identification), and 19/21 (90.5%; 95% CI 71.1–97.3%) met the stricter CI criterion with species or species complex designation. The *A. fumigatus* complex (n = 5) was correctly identified in all instances, with MyT scores ranging from 1.83 to 2.48 across replicate spots. *A. versicolor* complex isolates were reported at the complex level (*A. creber*/*A. sydowii*/*A. versicolor*), reflecting the limits of proteomic resolution for cryptic species within *Aspergillus* (discussed further in Section 4). One outlier isolate (reported originally as *Aspergillus* spp., non fumigatus) was consistently assigned to *A. montevidensis* (reclassified from *Eurotium amstelodami*); this identification was subsequently confirmed by the reference laboratory by sequencing, representing a genuine taxonomic reclassification rather than a misidentification (Section 4).

### 3.3. Other Hyaline Non-Aspergillus Moulds (n = 14)

Twelve of 14 isolates (85.7%; 95% CI 60.1–96.0%) achieved identification at ≥1.8. High confidence identifications (score ≥ 2.0) were obtained for *Lomentospora prolificans*, *Purpureocillium lilacinum*, *Byssochlamys spectabilis*, *Rasamsonia argillacea*, *Beauveria bassiana*, and *Sporothrix schenckii*. A *Fusarium lichenicola* isolate was assigned to the *F. solani* complex, consistent with documented molecular overlap within this species complex. Two *Penicillium* isolates yielded scores below 1.8 across all three spots; this appeared to coincide with dense conidiation at the colony edge at the time of harvest (interpretation in Section 4).

### 3.4. Mucorales (n = 8)

Seven of 8 Mucorales isolates (87.5%; 95% CI 52.9–97.8%) achieved species-level identification at the ≥1.8 threshold. *Mucor circinelloides*, *Cunninghamella bertholletiae*, *Lichtheimia corymbifera*, and *Rhizopus microsporus* were each correctly identified. One *Rhizopus arrhizus* isolate showed considerable variability across replicate spots (range 1.05–1.87); under the single highest scoring spot alone, this isolate would have returned a discordant identification, whereas the ≥2/3 concordance rule prevented this outcome (interpretation in Section 4). Given the rapid growth characteristics of Mucorales, Day 3 harvest and testing was necessary to avoid overgrowth artefact.

### 3.5. Dermatophytes (n = 8)

All 8 dermatophyte isolates (100%; 95% CI 67.6–100%) achieved overall identification at the ≥1.8 threshold, with 6/8 (75.0%; 95% CI 40.9–92.9%) meeting the stricter CI criterion. Resolution of intragroup species within the *T. mentagrophytes* complex, specifically, *T. indotineae* versus *T. mentagrophytes* sensu stricto, was not achievable with the current library version (clinical implications discussed in Section 4). The *T. rubrum* group, *Microsporum canis*, *T. tonsurans*, *Nannizzia gypsea*, and *Epidermophyton floccosum* were all correctly identified, with *M. canis* and *N. gypsea* achieving the highest absolute scores (2.22–2.43). Dermatophyte evaluation required a minimum of 96 h of incubation to yield sufficient leading-edge biomass.

### 3.6. Dematiaceous Fungi (n = 5)

Dematiaceous fungi represented the most challenging category evaluated, with identification at ≥1.8 achieved for only 2/5 isolates (40.0%; 95% CI 11.8–76.9%), while none of the isolates achieved species-level identification across ≥2/3 replicates. *Exophiala jeanselmei* scored below 1.8 by MyT across all replicate spots (highest score 1.18); parallel chemical extraction yielded a score of 1.75 in one replicate, approaching but not meeting the threshold. *Alternaria chartarum*, a *Bipolaris* isolate, and a *Cladosporium* isolate did not achieve concordant identification by either method. Given the small subgroup size (n = 5), this estimate should be interpreted with caution (interpretation and library-related context in Section 4).

### 3.7. Culture Medium Comparison: BHI Versus SAB-CG

BHI agar with 5% sheep blood, chloramphenicol, and gentamicin was associated with higher identification yield than Sabouraud with chloramphenicol and gentamicin (SAB-CG) across hyaline mould categories at Day 3. Among the 35 paired isolates evaluated, growth was observed in 31/35 (88.6%) isolates on BHI and 27/35 (77.1%) on SAB-CG. The paired outcomes were compared using an exact two-sided McNemar test. The reconstructed paired contingency table comprised 25 isolates that grew on both media, six that grew on BHI but not SAB-CG, two that grew on SAB-CG but not BHI, and two that failed to grow on either medium. The resulting exact McNemar *p*-value was 0.2891, indicating no statistically significant difference in growth between the two culture media.

### 3.8. Effect of Incubation Duration: Day 3 Versus Day 7

In this exploratory, post hoc comparison, Day 3 harvest outperformed Day 7 across the *Aspergillus* and other hyaline mould categories retested at both timepoints (n = 35), with an approximately four-fold difference in successful identification rate. By Day 7, aerial conidiation had progressed substantially in the majority of these colonies, producing a conidial-dominated surface zone whose spectra differed from vegetative-mycelium reference entries. For *Aspergillus fumigatus* specifically, mean MyT scores at Day 7 were 0.23 score units lower than at Day 3. These findings support 72 h as the preferred harvest timepoint for hyaline moulds within this workflow and are consistent with a shorter overall diagnostic timeline relative to conventional morphological maturation, which typically requires 5–7 days or longer. Day 3 identification was specifically relevant for our lab since processing of mycology samples is done Monday to Friday, and therefore, for the purpose of laboratory mycology work flow, it was very relevant for us to review Day 3 harvest-based identification.

### 3.9. Contribution of Triplicate Spotting

In a post hoc analysis restricted to the categories in which discordant single-spot results were observed (other hyaline non-*Aspergillus* moulds and Mucorales, combined n = 22), applying the ≥2/3 concordance rule at ≥1.8 prevented false identification calls that would otherwise have resulted from single high-scoring outlier spots in one Mucorales and two non-*Aspergillus* hyaline mould isolates (three isolates total). Conversely, the rule enabled identification of four further isolates for which a single spot fell below 1.8, but two of three spots scored at or above threshold with concordant identification, isolates that would have remained unidentified under a single-spot protocol. Reconstructing a single-spot equivalent outcome for this 22-isolate subgroup (19 triplicate consensus correct/acceptable identifications, minus the three corrected and four enabled by triplicate consensus) gives an estimated single-spot identification rate of 12/22 (54.5%), compared with 19/22 (86.4%) achieved with triplicate consensus, an approximately 32 percentage point absolute difference. This was a post hoc, hypothesis-generating analysis restricted to the affected subgroup and should not be extrapolated to the full 56-isolate panel or treated as a precise, generalisable effect size.

### 3.10. MyT Versus Chemical Extraction

For the 11 isolates tested in parallel by formic acid–ethanol extraction, MyT provided equivalent or higher scores in 8/11 (72.7%) instances (Table 2). Extraction offered a marginally higher score for two dematiaceous isolates and one Mucorales isolate, although both methods failed to reach the 1.8 threshold for these isolates. For all *Aspergillus* and hyaline mould isolates tested in parallel, MyT and extraction produced equivalent identification outcomes. MyT required substantially less hands-on time (approximately 5 min per isolate versus 25–30 min for extraction). This exploratory, underpowered comparison suggests MyT is a reasonable candidate as a frontline identification method for the most clinically prevalent categories tested here, with chemical extraction retained as a secondary option for isolates that fail MyT, particularly among dematiaceous fungi. Of note, only 11 isolates were tested in parallel; a formally powered non-inferiority comparison was not performed and would be needed before drawing firmer conclusions about comparability with extraction-based methods.

## 4. Discussion

Rapid and accurate identification of filamentous fungi remains a persistent challenge in routine clinical mycology laboratories. This study makes three main contributions to the clinical mycology literature. First, it provides a single-centre analytical validation of the Bruker MyT workflow, used as a standalone diagnostic platform, for filamentous fungi in a Canadian setting. A prior multi-library comparison by Stein et al. [14] benchmarked Bruker, MSI, and NIH libraries using conventional extraction protocols on an earlier library version; to our knowledge, this is among the first Canadian studies to evaluate the MyT method specifically, using a current library version. Second, this study provides an initial, exploratory estimate of the performance benefit of triplicate spotting with a ≥2/3 concordance rule at the 1.8 threshold within the MyT workflow: within the subgroup of categories affected by discordant single-spot results, the estimated 32 percentage point absolute gain over single-spot acquisition (Section 3.9) suggests that the acquisition strategy, and not only the sample preparation method, may be an important determinant of diagnostic yield. This modification requires no additional equipment, reagents, or specialised expertise and may be a reasonable target for laboratories seeking to improve mould identification performance within existing MALDI-TOF MS workflows, although this estimate should be confirmed in a larger, purpose-designed comparison. Third, the BHI versus SAB-CG comparison performed within the MyT protocol addresses a specific gap in the literature, as prior culture medium comparisons for MALDI-TOF MS mould identification were largely performed using conventional extraction protocols and may not be directly transferable to the MyT workflow. This is consistent with other recent work identifying culture medium and other pre-analytical variables as key determinants of MALDI-TOF MS filamentous fungi identification performance [10].

The overall concordance of 89.3% achieved using MBT Myco 4.1 alone is broadly consistent with the performance range reported in contemporary MALDI-TOF MS mould identification studies and compares favourably with earlier reports using single-library, single-spot protocols. Honsig et al. [8] reported identification rates of 48.9–76.1% across three extraction protocols using an older Bruker Filamentous Fungi Library version, with the highest rates achieved only with the technically demanding extraction method. The present MyT-based approach achieved comparable or higher performance without solvent extraction, which reflects both library version improvements and favourable spectral characteristics of leading-edge mycelium for several organism categories when acquisition is optimised. A 2025 multi-platform comparison by Klugherz et al. [5] reported valid identification rates of 71–81% across three MALDI-TOF MS systems, with the Bruker platform achieving the highest performance; our findings are consistent with the interpretation that the acquisition methodology, rather than the platform hardware alone, is a key determinant of mould identification yield. Direct comparison of the Bruker platform against an alternative commercial library (MSI-2) has similarly reported comparable filamentous fungi identification performance between systems when applied to liquid culture isolates [15], and testing algorithms that combine MALDI-TOF MS with reflex morphological or molecular confirmation have been reported to further improve overall diagnostic yield relative to single-modality approaches [16]. Extension of the reference library beyond the manufacturer default database has also been shown to improve identification yield in other clinical microbiology settings [17], reinforcing the library composition, rather than the underlying MALDI-TOF MS platform, as a principal determinant of performance for filamentous fungi.

*Aspergillus* species achieved 100% overall concordance, supporting the use of MyT for in house *Aspergillus* identification in the oncology and haematopoietic transplant population served by LHSC, with reduced need for external referral. The *A. versicolor* complex illustrates a known limit of proteomic taxonomy for closely related cryptic species: the spectral profiles of *A. versicolor*, *A. sydowii*, and *A. creber* are not reliably distinguished by mass spectrometry, and molecular sequencing of the calmodulin or benA genes remains the reference method for intracomplex resolution, consistent with previously described challenges specific to *Aspergillus* identification by MALDI-TOF MS [18]. For most clinical scenarios, complex-level identification is likely to be adequate, as susceptibility profiles within this complex are broadly similar.

*A. montevidensis* identification illustrates the distinction between MALDI-TOF MS misidentification and taxonomic reclassification. *Eurotium amstelodami* has been reclassified as *Aspergillus montevidensis* within the *Aspergillus* section *Aspergillus* based on multilocus phylogenetic analyses; the spectral library reflects current taxonomy, whereas the original morphology-based identification predated this nomenclatural revision. The apparent discrepancy therefore represents taxonomic evolution rather than analytical error. This case underscores a practical advantage of MALDI-TOF MS: unlike conventional morphological identification, which depends on subjective interpretation and static taxonomic reference points, proteomic identification leverages periodically updated reference databases that can incorporate advances in fungal systematics over time.

For the two *Penicillium* isolates that did not achieve identification (Section 3.3), dense conidiation at the colony edge at the time of harvest may have generated spectra that differ from the vegetative mycelium reference entries in the library; a repeat-sampling approach targeting a less-conidiated region of the colony may merit evaluation before recording a no-identification outcome for such isolates in routine practice.

The Mucorales performance of 87.5% overall identification is of particular clinical relevance, as species-level identification within this order can influence both the therapeutic approach and the prognosis. *Rhizopus arrhizus*, the most common agent of mucormycosis, is generally more susceptible to posaconazole and isavuconazole than *Cunninghamella bertholletiae*, which is associated with higher case fatality and treatment failure rates [1,19]. The ≥2/3 consensus rule prevented a false identification call on a variable *Rhizopus arrhizus* isolate in this panel (Section 3.4), illustrating a specific safety benefit of multi-spot acquisition for this order.

Dermatophyte performance of 100% overall concordance supports the use of MyT for routine dermatophyte identification at LHSC. The inability to distinguish *T. indotineae* from *T. mentagrophytes* sensu stricto is of increasing clinical importance, as *T. indotineae* carries SQLE mutations conferring high-level terbinafine resistance and has been documented in Canada and should be considered in patients presenting with treatment refractory tinea or with epidemiological links to South Asia [20,21]. A recent multicentre evaluation of commercial, in-house, and web-based MALDI-TOF MS databases similarly reported that species-level resolution within the *T. mentagrophytes* group ranged from only 30.0% to 78.9%, depending on the database used, with combination of commercial and in-house databases improving performance [20], consistent with our finding that group-level identification, but not intragroup discrimination, was achievable with the current library version. Until library updates enable intrinsic discrimination of *T. indotineae*, group-level *T. mentagrophytes* identifications may warrant supplementary SQLE sequencing or terbinafine susceptibility testing in patients with treatment refractory tinea or relevant epidemiological risk factors.

The poor performance of dematiaceous fungi is the one category in this validation where MyT alone, even with optimised triplicate-spot acquisition, did not achieve an adequate diagnostic yield. This reflects a known structural limitation of current spectral databases rather than a failure of the MyT methodology itself: melanised moulds remain underrepresented in library construction efforts historically driven by *Aspergillus*-centric clinical demand, and melanin pigments may physically interfere with the ionisation process, yielding systematically lower spectral signal quality [9,22]. Given the small subgroup size evaluated here (n = 5), this finding should be treated as preliminary; in-house library expansion may be a reasonable approach to closing this specific gap for the melanised mould species relevant to the local isolate population [23].

The relevance of this work extends beyond laboratory methodology to the core clinical problem motivating it: diagnostic delay. Diagnostic delay in filamentous fungal identification has been highlighted as a contributor to excess morbidity and mortality in invasive fungal disease, and to inefficient antifungal use [3]. In our institutional experience, a 10–14 calendar day identification delay for organisms whose recognition can immediately reshape antifungal selection represents an area where diagnostic workflow changes may have a measurable clinical impact, although this study did not directly measure turnaround time or clinical outcomes. Species-level identification has concrete potential implications for antifungal selection: *Aspergillus terreus*, correctly identified in this panel, is intrinsically resistant to amphotericin B [24], and recognising this organism could support an earlier switch from liposomal amphotericin B to voriconazole or isavuconazole. Similarly, identification of *Lomentospora prolificans*, which is intrinsically resistant to most routinely tested antifungal agents, could prompt earlier escalation to salvage combination therapy and infectious diseases consultation. Distinguishing *Cunninghamella bertholletiae* from *Rhizopus arrhizus* has potential implications for azole selection, given differential susceptibility [19]. We emphasise that these are anticipated downstream benefits of accurate, timely identification rather than outcomes measured in this study.

A systematic review of diagnostics-driven antifungal stewardship interventions reported that MALDI-TOF MS integration was associated with reductions in antifungal consumption of 11.6–59.0% and cost savings of 13.5–50.6% across included studies, without evidence of adverse effects on mortality [25]. We cite this literature as context and motivation rather than as a result of the present study: whether in-house MyT identification at LHSC would translate into comparable turnaround time, stewardship, or cost benefits has not been evaluated here and is proposed as a specific direction for future prospective work (Section 5) [5,26].

Beyond incremental optimisation of the MyT workflow itself, proteomic fungal identification is increasingly moving toward integration with computational and machine learning tools; we discuss this, together with other future directions, in Section 5.

Real-world experience outside dedicated validation studies illustrates the performance ceiling of routine MALDI-TOF MS for filamentous fungi under typical operating conditions. A retrospective Australian series reported successful identification in 66.1% of attempted filamentous fungal isolates over a five-year period [27], lower than the concordance achieved in the present optimised, triplicate-spot validation. This gap is consistent with the interpretation that acquisition strategy and library recency, rather than instrumentation alone, are important determinants of real-world diagnostic yield, and suggests that the gains demonstrated here may not be fully realised without disciplined triplicate acquisition and threshold adherence in routine practice.

Beyond individual patient care, in-house mould identification could support institutional fungal epidemiological surveillance, including ongoing monitoring of *Aspergillus* section-level epidemiology, Mucorales species distribution, and the emergence of antifungal-resistant dermatophytes within the LHSC population, which in turn could inform antifungal prophylaxis strategies, environmental surveillance, and infection prevention resource allocation. These applications were not evaluated in the present study and are proposed as directions for future work.

## 5. Limitations and Future Directions

This study has several limitations. The total panel of 56 isolates, while adequate for an overall concordance estimate, is small for formal validation of individual fungal categories, particularly dematiaceous fungi (n = 5) and specific Mucorales subgroups, as reflected in the wide confidence intervals around these subgroup estimates. With the exception of *A. fumigatus* and *A. niger*, most individual species and species complexes in this panel are represented by fewer than the three isolates per species that would ideally be required for a robust species-level validation, and several species are represented by only a single isolate; the species-level findings should therefore be regarded as preliminary, with category-level concordance as the primary performance measure of this study.

The use of archived isolates rather than prospectively collected fresh clinical specimens may introduce selection bias relative to isolates encountered in routine practice, and reference identifications were not uniformly confirmed by molecular sequencing, which could inflate apparent concordance for taxa in which morphology-based reference identification is itself imperfect. All testing was performed at a single centre; a formal inter-operator and inter-site reproducibility study was not performed, and this is a specific priority for future work on MyT sampling, since harvesting the leading colony edge by hand is inherently operator dependent. The extraction comparison was performed in only 11 isolates and was not powered to detect modest performance differences between methods. The triplicate- versus single-spot comparison (Section 3.9) and the Day 3 versus Day 7 comparison (Section 3.8) were post hoc, exploratory analyses defined after data collection; these results should be interpreted as hypothesis generating rather than confirmatory, and formal correction for multiple comparisons was not applied given the exploratory nature of the subgroup analyses.

Planned future work includes expansion of the validated isolate panel, particularly for dematiaceous fungi, rare Mucorales species, and underrepresented non-fumigatus *Aspergillus* species and other individual species currently represented by small numbers, together with targeted in-house spectral library expansion; a formal, adequately powered comparison of MyT with chemical extraction and with single-spot acquisition; and a prospective evaluation of turnaround time and clinical outcomes following implementation, alongside a formal health economic analysis comparing in-house MyT identification with the current reference laboratory send-out model. In bacteriology, large curated spectral repositories such as the Database of Resistance Information on Antimicrobials and MALDI-TOF Mass Spectra (DRIAMS) have enabled machine learning models that predict antimicrobial resistance directly from whole-cell spectra, in addition to organism identification [28]. Comparable open, multicentre spectral datasets specifically for filamentous fungi remain limited, although recent initiatives to share routine MALDI-TOF MS datasets that include fungal isolates may help address this gap and could, in principle, support similar machine learning approaches for mould identification and resistance prediction in the future [29]. Prospective evaluation of machine learning-assisted spectral analysis, and contribution of locally generated spectral data to shared, multicentre fungal MALDI-TOF MS repositories, represent additional avenues that could further improve identification yield and support reference library currency over time [28].

## 6. Conclusions

This study provides a single-centre analytical validation of a triplicate-spot Bruker MALDI-TOF MS MyT workflow as a standalone identification platform for clinically relevant filamentous fungi, with 89.3% overall concordance and zero misidentifications across a 56-isolate panel. In an exploratory, post hoc analysis restricted to the categories affected by discordant single-spot results (a 22-isolate subgroup, not the full 56-isolate panel), triplicate spotting with a ≥2/3 concordance rule was associated with an estimated 32 percentage point improvement in identification relative to single-spot acquisition within that subgroup, and 72 h incubation was identified as the preferred harvest timepoint for hyaline moulds within this workflow.

These findings support MyT as a practical identification method for *Aspergillus* species, Mucorales, and dermatophytes using existing diagnostic infrastructure, with dematiaceous fungi and several individual, small “n” species remaining categories of limited or uncertain performance under the current library coverage and panel size. Potential downstream benefits such as reduced turnaround time, antifungal stewardship gains, and cost savings are plausible, based on the broader literature, but were not measured in this study and should be regarded as hypotheses for prospective evaluation rather than demonstrated outcomes. For laboratories serving comparable high-risk immunocompromised populations, this validation may provide a useful reference framework for implementation planning, while appropriate local verification remains advisable given inter-laboratory differences in isolate populations and operational conditions.

## Figures and Tables

**Table 1:** MALDI-TOF MS MyT identification performance by fungal category (triplicate-spot consensus, ≥1.8 threshold).

Fungal Category	Isolates (n)	CI ≥ 1.8 (n)	CI %	Overall ID ≥ 1.8 (n)	Overall ID %	No ID (n)	Misidentified (n)
*Aspergillus* spp.	21	19	90.5%	21	100.0%	0	0
Other hyaline moulds (non-*Aspergillus*)	14	11	78.6%	12	85.7%	2	0
Mucorales	8	7	87.5%	7	87.5%	1	0
Dermatophytes	8	6	75.0%	8	100.0%	0	0
Dematiaceous fungi	5	0	0.0%	2	40.0%	3	0
Total	56	43	76.8%	50	89.3%	6	0

CI: correct identification at species or species-complex level. Overall ID: CI + acceptable genus-level identification. No ID: all three spots scored <1.8. Wilson 95% confidence intervals for the panel totals are reported in the text (Section 3.1).

**Table 2:** Triplicate MyT score performance and comparison with chemical extraction.

Organism (n)	Average MyT Spot 1	Average MyT Spot 2	Average MyT Spot 3	Average Extraction Score	MyT Identification
*A. fumigatus* (n = 4)	2.06	1.93	2.04	2.23	Correct (4/4)
*A. versicolor* complex (n = 2)	2.19	2.21	2.40	—	Correct (2/2)
*A. niger* (n = 2)	2.27	2.01	2.39	2.23	Correct (2/2)
*Mucor circinelloides* (n = 2)	1.80	2.03	1.98	1.57	Correct (2/2)
*Rhizopus* spp. (n = 3)	1.92	1.85	1.94	—	Correct (2/3)
*T. mentagrophytes* group (n = 2)	2.28	2.20	2.16	—	Correct (2/2)
*T. rubrum* group (n = 1)	npf	2.32	2.32	—	Correct (1/1)
*Fusarium* spp. (n = 3)	1.81	1.98	1.96	—	Correct (2/3)
*Scedosporium/Lomentospora* (n = 2)	1.90	2.18	2.29	—	Correct (2/2)
*Sporothrix schenckii* (n = 2)	1.89	2.01	1.99	2.01 ± 0.02	Correct (2/2)
*Exophiala jeanselmei* (n = 1)	1.18	1.17	1.06	1.75	No ID
*Beauveria bassiana* (n = 2)	1.91	1.89	2.02	—	Correct (2/2)

Scores expressed as mean (n > 1) or individual score (n = 1); npf: no peaks found. Species-level identification required ≥2/3 spots at ≥1.8.

## Data Availability

The original contributions presented in this study are included in the article. Further inquiries can be directed to the corresponding author.
